# Association Between HLA Alleles and IgA Nephropathy in a Taiwanese Population

**DOI:** 10.3390/ijms27020790

**Published:** 2026-01-13

**Authors:** Yung-Chieh Huang, I-Chieh Chen, Guan-Cheng Lin, Tzu-Hung Hsiao, Shang-Feng Tsai, Yi-Ming Chen, Lin-Shien Fu

**Affiliations:** 1 Division of Nephrology, Department of Pediatrics, Taichung Veterans General Hospital, Taichung 407, Taiwan; 2 Department of Post-Baccalaureate Medicine, College of Medicine, National Chung Hsing University, Taichung 402, Taiwan; 3 Doctoral Program in Translational Medicine, National Chung Hsing University, Taichung 402, Taiwan; 4 Department of Medical Research, Taichung Veterans General Hospital, Taichung 407, Taiwan; 5 Institute of Genomics and Bioinformatics, National Chung Hsing University, Taichung 402, Taiwan; 6 Division of Nephrology, Department of Internal Medicine, Taichung Veterans General Hospital, Taichung 407, Taiwan; 7 Division of Allergy, Immunology and Rheumatology, Department of Internal Medicine, Taichung Veterans General Hospital, Taichung 407, Taiwan; 8 School of Medicine, National Yang Ming Chiao Tung University, Taipei 112, Taiwan

**Keywords:** HLA, IgA nephropathy, Taiwan, single-nucleotide polymorphism

## Abstract

Genetic associations with IgA nephropathy (IgAN), particularly in the human leukocyte antigen (HLA) region, vary across ethnic groups. This study investigated the association of HLA alleles with the diagnosis, pathological findings, and prognosis of biopsy-proven IgAN in a Taiwanese population. A case-control study was conducted using data from the Taiwan Precision Medicine Initiative, including 157 patients with biopsy-proven IgAN and 1570 age- and sex-matched controls. Genetic data were obtained from single-nucleotide polymorphism arrays, and HLA imputation was performed. Most single-nucleotide polymorphisms associated with IgAN were located within the HLA region on chromosome 6. Frequencies of several alleles (including C*08:01, DQA1*03:01, and DQB1*04:01) were significantly higher in the IgAN group. Conversely, frequencies of alleles such as B*58:01 and DQB1*02:01 were significantly lower. This study identified novel risk and protective HLA alleles for IgAN in a Taiwanese population.

## 1. Introduction

In Taiwan, immunoglobulin A (IgA) nephropathy (IgAN) is the most prevalent form of primary glomerulonephritis (GN), accounting for 26% of all primary GN cases according to the National Renal Biopsy Registry in Taiwan [1]. Globally, and particularly in East Asia, IgAN is recognized as the most common primary GN [2,3], although its incidence varies substantially among different ethnic groups [1,4,5]. Despite its prevalence, the pathophysiology of IgAN remains incompletely understood [6,7]. The prevailing model, known as the multi-hit hypothesis [2], suggests that the disease involves several factors, including abnormal galactosylation of IgA1, a mucosal immunity response, activation of both innate and adaptive immunity, and the formation and deposition of IgA-containing immune complexes.

Numerous studies have explored the association between IgAN and specific single-nucleotide polymorphisms (SNPs) [8,9,10,11,12,13,14,15,16,17,18,19,20,21,22,23,24,25,26,27,28,29,30], with a notable focus on SNPs in the human leukocyte antigen (HLA) region [30,31,32,33]. Various HLA alleles have been implicated in IgAN [34,35,36,37]. For example, HLA-DRB1*15:01, DQB1*02:02, DQB1*03:02, and DQB1*04:01 were associated with IgAN in a Korean patient cohort [34]. In Europe, a decreased frequency of DQB1*0201 was observed in British patients with IgAN, whereas Finnish patients exhibited a decreased frequency of DQB1*0602 [35]. A study conducted over 30 years ago by Huang and colleagues investigated the relationship between HLA and IgAN in Taiwan but found no significant association with either IgAN or prognostic markers [38]. This lack of association was possibly due to the relatively small sample size and the low resolution of HLA typing at the time.

The clinical presentation of IgAN can vary considerably between individuals, ranging from asymptomatic cases with microscopic hematuria and stable renal function to instances of rapid renal function decline or early progression to end-stage kidney disease (ESKD). Many studies [34,39,40] have indicated that specific HLA alleles or genetic risk scores (GRSs) are associated with IgAN progression. However, these findings have not yet been widely integrated into clinical practice.

With advancements in DNA sequencing technologies over the past decades, higher-resolution HLA typing and genome-wide association studies (GWASs) have largely supplanted traditional HLA serotyping [41]. Therefore, this study aimed to investigate the association of biopsy-proven IgAN with both SNPs and HLA alleles in a Taiwanese population. Furthermore, t We sought to determine the association between specific HLA alleles and the pathological findings and the prognosis of IgA nephropathy.

## 2. Results

### 2.1. Participant Characteristics and Pathological Data

A total of 157 patients with biopsy-proven IgAN were included in the analysis, alongside 1570 participants in the control group. The baseline characteristics and biochemical data of the IgAN and control groups are presented in Table 1. Of the patients with IgAN, 101 had a MEST-C score according to the Oxford Classification, and 117 had an adequate immunofluorescence report. The pathological findings for the patients with IgAN are listed in Table 2; all the patients had IgA deposit 2+ or more.

#### 2.1.1. Association of HLA Alleles with IgAN Susceptibility

Then, we performed genome-wide imputation for all study participants; however, the present analysis specifically focused on eight classical HLA loci (HLA-A, HLA-B, HLA-C, HLA-DPA1, HLA-DPB1, HLA-DQA1, HLA-DQB1, and HLA-DRB1) to investigate the association between HLA allele carrier status and IgAN.

**Figure 1:**
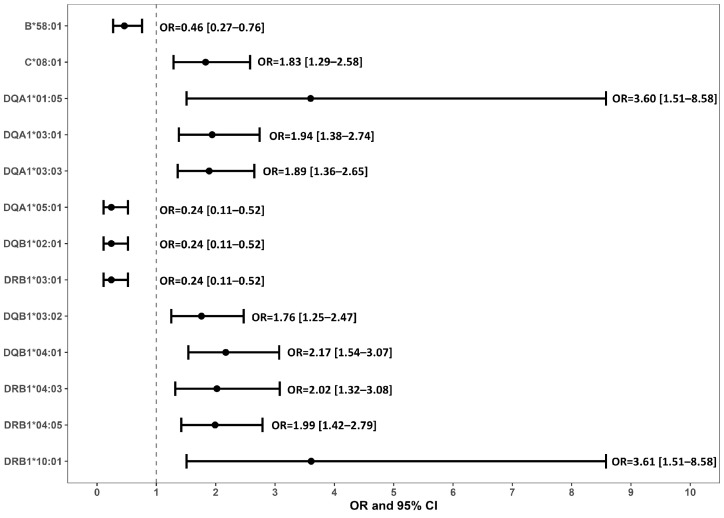
Forest plot of odds ratios (ORs) and 95% confidence intervals for HLA alleles significantly associated with IgAN.

We examined the genotype frequencies of HLA alleles in the IgAN and control groups, and the results are summarized in Table 3 and Figure 1.

After adjusted for age, sex, hypertension, diabetes, and hyperlipidemia, and were corrected for multiple testing using the BH-FDR method, the risk of HLA-C*08:01, DQA1*01:05, DQA1*03:01, DQA1*03:03, DQB1*03:02, DQB1*04:01, DRB1*04:03, DRB1*04:05, and DRB1*10:01 were significantly higher in patients with IgAN than in controls.

Conversely, HLA-B*58:01, DQA1*05:01, DQB1*02:01, and DRB1*03:01 exhibited significantly lower risk in patients with IgAN compared with the control group.

#### 2.1.2. Distribution of HLA Allele Genotypes by Pathological Features

We further examined the genotype frequencies of HLA alleles in patients with IgAN according to their pathological findings from renal biopsies (Table 4). Among the 101 biopsy-confirmed IgAN cases, MEST-C scores and HLA allele typing data were available for 100 patients. In the univariate analysis, the frequency of HLA-DQA1*03:01 was significantly higher in patients with segmental sclerosis (*p* = 0.0202). Although HLA-DQB1*03:02 was more frequent in cases with segmental sclerosis, this difference did not reach statistical significance (*p* = 0.0502). In a supplementary multivariable analysis (Supplementary Table S2), we further evaluated the association between HLA alleles and the presence of segmental glomerulosclerosis after adjusting for age, sex, hypertension, diabetes, and hyperlipidemia. None of the HLA alleles remained statistically significant after FDR correction. The alleles DQA1*01:05 and DRB1*10:01 were not analyzed because no carriers were present in the S0 (non-segmental sclerosis) group.

#### 2.1.3. Analysis of HLA Alleles and Renal Prognosis

Of the patients with IgAN, 62 experienced progression to ESKD by their most recent follow-up, whereas 24 patients had early CKD. No significant differences in HLA AFs were found between the patients without versus with ESKD nor between those without ESKD and those with ESKD plus early CKD (Supplementary Table S3).

We further compared the HLA alleles showing significant associations in our study with those previously reported in other populations. As summarized in Table 5, several alleles, such as DQB103:02*, DRB104:03*, and DRB104:05*, have been consistently identified as risk alleles across multiple ethnic cohorts.

However, we also identified HLA-C*08:01, DQA1*01:05, DQA1*03:01, DQA1*03:03, DQB1*04:01, and DRB1*10:01 as novel risk alleles associated with IgAN in our cohort. Conversely, HLA-B*58:01 and DQA1*05:01 were found to be protective alleles that have not been previously reported in other studies.

## 3. Discussion

Our study revealed that the AFs of HLA-C*08:01, DQA1*01:05, DQA1*03:01, DQA1*03:03, DQB1*03:02, DQB1*04:01, DRB1*04:03, DRB1*04:05, and DRB1*10:01 were significantly higher among patients with IgAN, whereas those of HLA-B*58:01, DQA1*05:01, DQB1*02:01, and DRB1*03:01 were significantly lower. These findings suggest a potential role for adaptive immunity in the pathogenesis of IgAN. HLAs, or MHC molecules, play critical roles in antigen processing and presentation in adaptive immunity. MHC molecules are involved in the regulation of intestinal inflammation and IgA production and may influence the antiglycan response [44]. The role of HLA in IgAN pathogenesis may be similar to that in other autoimmune diseases associated with IgA deficiency, although the precise mechanism remains incompletely understood [45].

Several SNPs located within the HLA region have been reported to be associated with IgAN. Feehally and colleagues identified rs3115573 and rs3130315 as the 2 SNPs with the strongest association, along with 3 SNPs (rs13209234, rs11244, and rs2857106) for which the conditional association is significant, in a cohort from the UK Glomerulonephritis DNA Bank [31]. In a study by Yu and colleagues involving a Chinese cohort, 3 SNPs (rs660895, rs1794275, and rs2523946) in the HLA region were associated with IgAN. Another Chinese cohort study [33] revealed that rs3077, rs9277535, and rs7453920 in the HLA-DP/DQ region were associated with IgAN. Additionally, a European and UK study on IgAN GRSs [40] included 4 SNPs in the HLA-DR and HLA-DQ region (rs7763262, rs9275224, rs2856717, and rs9275596) in its risk score calculation for IgAN prevalence in these populations. In our analysis, we identified 26 SNPs in the HLA region that achieved genome-wide significance. Future research is required to determine whether specific HLA alleles, SNPs, or a polygenic risk score could serve as diagnostic or prognostic tools for IgAN.

Currently, renal biopsy remains the sole method for confirming an IgAN diagnosis; however, the risk of bleeding-related complications associated with the procedure has been reported to be between 8 and 34% [46,47,48]. Although genetic diagnosis cannot yet replace renal biopsy, it holds potential as an alternative diagnostic method, offering a lower risk of patient harm compared with biopsy.

Of the HLA alleles identified in the present study, DQB1*03:02 [32], DRB1*04:03 [43], and DRB1*04:05 [34,43] have previously been reported as risk factors for IgAN. The OR of 1.42 forDQB1*03:02reported by Yu and colleagues is comparable to our finding of an OR of 1.76 (95% CI = 1.25–2.47). DRB1*04:03 was reported to have an OR of 1.318 by Yang and colleagues, which is lower than the OR found in our study (OR = 2.02, 95% CI = 1.32–3.08). The OR of DRB1*04:05 was 1.99 (95% CI = 1.42–2.79) in our study, similar to that reported by In and colleague (OR = 1.76) but higher than that reported by Yang and colleagues (OR = 1.267). Conversely, DQB1*02:01, which was found to be protective in our patient group, was also found to have a decreased frequency among British patients but not among Italian or Finnish patients in a European study [35].

In addition to these similarities, we identified HLA-C*08:01, DQA1*01:05, DQA1*03:01, DQA1*03:03, DQB1*04:01, and DRB1*10:01 as novel risk alleles associated with IgAN, although these alleles have not been reported as significant in other studies. Additionally, HLA-B*58:01 and DQA1*05:01, identified as protective in our study, have not been previously reported with to have similar protective effects. Variations in genetic backgrounds and the relatively small sample size in our study may account for the differences in our results compared with those of other studies. These findings are consistent with the comparative summary presented in Table 5.

Specific HLA alleles have also been reported to be associated with ESKD in patients with IgAN. An early study linked ESKD to with HLA DQB1*0301 in patients with IgAN [42]. In a Korean cohort, the DQB1*05:03 allele was more common in individuals without ESKD than in those with ESKD [34]. Conversely, a study conducted on a Chinese population by Shi and colleagues [14] did not report a significant association between HLA-DQ/DR alleles and ESKD Consistent with these latter findings, we did not identified any significant positive or negative associations between HLA alleles and ESKD or, more broadly, early CKD. Our findings can be interpreted in the context of the current understanding of IgAN pathophysiology, the multi-hit hypothesis [49]. As our data strongly suggest, HLA molecules are fundamental to the adaptive immune response. We propose that the risk-associated HLA alleles identified in our study (e.g., HLA-C*08:01, DQA1*03:01) create a genetic susceptibility that facilitates the initial pathogenic events, such as the production of galactose-deficient IgA1 (Gd-IgA1) and the subsequent auto-antibody response. This explains their strong association with the risk of developing IgAN in the first place. However, the progression from IgA deposition to severe renal damage (fibrosis, sclerosis, and ESKD) is a distinct downstream process. This phase is likely governed by a different set of factors, such as the intrinsic renal response to injury, complement activation, podocytopathy, and tubulointerstitial inflammation. It is therefore biologically plausible that the genetic factors governing initiation (HLA) are different from those governing progression.

Our results reinforce the ‘two-stage’ hypothesis. While risk-associated HLA alleles (e.g., HLA-C*08:01, DQA1*03:01) clearly contribute to disease susceptibility, their role in subsequent disease progression appears not significant. We specifically investigated the link between HLA alleles and pathological features. In our initial univariate analysis, DQA1*03:01 showed an association with segmental sclerosis. However, this association did not remain significant after adjusting for key clinical confounders (age, hypertension, diabetes, etc.) in a robust multivariable model (Supplementary Table S2). This finding strongly suggests that pathological progression (such as the development of ‘S’ lesions) is likely driven by non-HLA factors—such as the intrinsic renal response to injury, hypertension, or metabolic factors—rather than being directly determined by the HLA genotype itself. This clarifies the “two-stage” model, confining the primary role of HLA to disease initiation.

Beyond the HLA region, several loci in our analysis showed suggestive associations with IgAN, although they did not reach genome-wide significance. These loci may still play a biological role in disease susceptibility, particularly those related to immune regulation, glycosylation pathways, and kidney structural integrity. For instance, SLC2A2 encodes a glucose transporter that could indirectly influence glycan metabolism and immune complex formation, both of which are central to IgAN pathogenesis. Further genome-wide analyses with larger cohorts and fine-mapping are warranted to validate these potential non-HLA associations and clarify their biological implications.

To the best of our knowledge, this is the first study to analyze the association between genetic variants and specific kidney pathology features in IgAN. The MEST-C scoring system in the Oxford Classification of IgAN was developed for assessing a patient’s risk and making a prognosis [50]. However, a recent validation study demonstrated inconsistent correlations between MEST-C scores and outcomes [51]. Ray and colleagues [52] reported a case involving a patient with a homozygous high-risk G1 *APOL1* variant, presenting with CKD stage 3A, microscopic hematuria, and subnephrotic range proteinuria. Kidney biopsy of this patient revealed both IgAN and focal segmental glomerulosclerosis. Apart from this case report, segmental sclerosis in IgAN has not previously been associated with specific genetic variants. Although we did not find significant HLA alleles influencing the patient’s long-term prognosis, our robust multivariable analysis also did not confirm a significant independent link between HLA alleles and key pathological findings (such as segmental sclerosis). In addition, potential non-HLA variants identified in our study warrant further investigation to elucidate their contribution to IgAN susceptibility and progression.

This study has several limitations. First, the relatively small number of biopsy-confirmed IgAN cases in this single-center retrospective study may have limited the statistical power to detect modest associations, and some findings may not have reached statistical significance; replication in larger independent cohorts will be essential. Additionally, the follow-up duration for the patients with IgAN may not have been sufficient to observe the development of ESKD. Genotyping was conducted using the TWBv2 SNP array, which may have missed other potentially relevant genetic variants. Furthermore, because all the participants in this study were Taiwanese, the applicability of our findings to other ethnic groups is limited, though some HLA alleles that were identified are consistent with those reported in other studies. Although the TPMI control cohort was drawn from hospital-based participants rather than strictly healthy volunteers, this approach has been widely used in genetic studies of IgAN [32,34]. The genetic background of Taiwan’s population is heterogeneous, including subpopulations such as Chinese-Han (Minnan, Hakka, and Mainlander) and Austronesian groups [41,53]. Consequently, the associations between HLA AFs and diseases observed in this study may differ from those in neighboring countries [54].

## 4. Materials and Methods

### 4.1. Study Design and Data Source

This was a retrospective study based on data from the Taiwan Precision Medicine Initiative (TPMI), a nationwide precision medicine program launched by Academia Sinica in collaboration with 16 healthcare systems and 33 hospitals across Taiwan [55,56]. Although TPMI provides a countrywide framework for genomic and clinical data collection, the present analysis was restricted to participants recruited at Taichung Veterans General Hospital (TCVGH), a tertiary medical center in central Taiwan and one of the key participating institutions in the TPMI.

This study was conducted in accordance with the Declaration of Helsinki. The study protocol was reviewed and approved by the Institutional Review Board of Taichung Veterans General Hospital (IRB no. CE23454A). All participants provided written informed consent as part of the TPMI project.

### 4.2. Patients

Patients with renal biopsy-proven IgA nephropathy (IgAN) were identified from the TPMI–TCVGH cohort and designated as the IgAN group. The inclusion criteria were: (1) age ≥ 18 years at the time of renal biopsy; and (2) a clinical follow-up duration of more than one year. Participants from the TPMI–TCVGH cohort without IgAN were randomly selected as the control group and matched by sex and age in a 1:10 ratio to the IgAN group. A subset of patients (*n* = 40) had their biopsy-proven IgAN diagnosis confirmed at external facilities prior to enrollment; while their diagnosis was verified, their detailed pathological and immunofluorescence reports were not available for central review.

Individuals with suspected IgAN but lacking a renal biopsy were excluded from both groups. Additionally, patients with Henoch–Schonlein purpura, systemic lupus erythematosus, or chronic liver disease [31] were also excluded from both groups. The flow diagram of study participant selection is presented as Supplementary Figure S1.

We used the MEST-C score based on the Oxford Classification of IgAN [50] for our analysis. This classification system defines five prognostic features in renal biopsies: mesangial hypercellularity (M), endocapillary hypercellularity (E), segmental sclerosis (S), interstitial fibrosis/tubular atrophy (T), and cellular crescents (C) [57]. To ensure diagnostic accuracy and inter-rater reliability, all renal biopsy slides were independently reviewed by two experienced nephropathologists. Any discrepancies in scoring were presented and resolved by consensus at a specialist review meeting.

The estimated glomerular filtration rate (eGFR) of the participants was calculated using their serum creatinine level and the CKD-EPI equation. IgAN progression was categorized into ESKD or early chronic kidney disease (early CKD). In this analysis, ESKD was defined as an eGFR of <15 mL/min/1.73 m^2^ or the requirement for dialysis or having undergone renal transplant. Early CKD was defined as an eGFR of <60 mL/min/1.73 m^2^ before the age of 40 years [58,59].

### 4.3. Genotyping and Quality Control

Genomic DNA was isolated from 58,091 participants in the TPMI project using DNA isolation kits from TIANGEN Biotech (Beijing, China). Subsequently, the concentration of the DNA samples was measured using a NanoDrop 2000 Spectrophotometer (NanoDrop Technologies, Wilmington, DE, USA). Genotyping was performed using the Axiom Genome-Wide TWB 2.0 Array Plate, developed by Affymetrix (Santa Clara, CA, USA). This array plate, designed specifically for the Han Chinese population in Taiwan, covers 714,431 SNPs [60]. Following genotyping, the data were rigorously analyzed and subjected to quality control using Affymetrix Power Tools software. Software was accessed from Thermo Fisher Scientific (https://www.thermofisher.com/tw/zt/home/technical-resources/software-downloads/axiom-analysis-suite.html) on 1 July 2025. Markers that did not meet the stringent criteria were excluded. The criteria for exclusion included failure in the Hardy–Weinberg equilibrium tests with a *p* value of <1.0 × 10^−5^, a minor allele frequency (MAF) of <0.01, or a total MAF of <0.01. After these quality control measures, 591,048 SNPs remained and were used for subsequent analysis [54]. For statistical genetic analyses and genetic data quality control, the PLINK 1.9 software package was employed [61].

#### 4.3.1. HLA Allele Typing (Sequencing-Based HLA Genotyping)

Our study included 58,091 participants, from whom genomic DNA was extracted from 2 mL of peripheral venous blood. Genome-wide genotyping was performed using the customized Axiom™ Taiwan Precision Medicine Array (Thermo Fisher Scientific, Waltham, MA, USA). HLA alleles were inferred based on linkage disequilibrium patterns between single nucleotide polymorphisms (SNPs) across the extended major histocompatibility complex (MHC) region using the Axiom™ HLA Analysis 1.2 software (Thermo Fisher Scientific, Waltham, MA, USA). This approach enables high-resolution HLA typing through statistical imputation rather than direct sequencing.

#### 4.3.2. Quality Control of HLA Imputation

For participants without directly typed HLA alleles, we performed HLA imputation using SNP genotype data extracted from the extended major histocompatibility complex (xMHC) region. Imputation was conducted using SNP genotypes from the TWBv2 array, specifically with the R package HIBAG (HLA Genotype Imputation with Attribute Bagging) (available at https://bioconductor.org/packages/HIBAG, accessed on 1 July 2025). [62]. The procedure followed the methodology established for the Taiwanese population by Huang et al. [41]. The reference panel was derived from the Taiwan Biobank, integrating high-resolution HLA sequencing data with SNP array genotypes from over one thousand individuals, providing imputation models optimized for East Asian populations.

For each sample, imputed two-field (four-digit) HLA alleles were retained if the posterior probability was ≥ 0.9. The mean call rate across loci was 98.1%, and the imputation accuracy for the TWBv2 array, as reported by Huang et al., exceeded 96% for all loci at the two-field level. In our dataset, a random subset of individuals (*n* = 100) with both directly genotyped and imputed HLA data was used to assess concordance, showing > 97% agreement (Supplementary Table S1).

The imputation targeted three HLA genes (HLA-DQA1, HLA-DQB1, and HLA-DRB1) and provided two-field (four-digit) resolution for alleles with frequencies ≥ 5.0%. Imputed alleles were retained for analysis only if their posterior probability met the quality control threshold (CT ≥ 0.5). Downstream analyses, including association testing between HLA alleles and IgA nephropathy (IgAN) risk, were conducted based on allele carrier status. Only alleles with a frequency ≥ 5% and high imputation quality were included in the statistical analyses.

### 4.4. Statistical Analysis

Statistical analyses were conducted using SPSS (version 22.0; IBM Corp., Armonk, NY, USA). Categorical variables between two groups were compared using the chi-square test and are presented as numbers (percentages). Continuous variables were compared using Student’s *t* test and are presented as means and standard deviations (SDs). For genetic association analyses, univariable and multivariable logistic regression models were used to estimate the associations between HLA allele carrier status and the risk of IgA nephropathy (IgAN). Multivariable models were adjusted for age, sex, hypertension, diabetes, and hyperlipidemia. Odds ratios (ORs), 95% confidence intervals (CIs), and *p*-values were reported.

To account for multiple testing across HLA alleles in the regression analyses, *p*-values were corrected using the Benjamini–Hochberg false discovery rate (FDR) method. For analyses that involved only a single statistical comparison (e.g., associations between HLA alleles and clinical or pathological characteristics), the chi-square test was applied and no multiple-testing correction was required. A two-sided *p*-value < 0.05 was considered statistically significant.

## 5. Conclusions

To the best of our knowledge, this is the first study to investigate IgAN in a Taiwanese population by using contemporary genetic analysis techniques. Our findings reveal that the majority of SNPs associated with IgAN are located in the HLA region. Further analysis indicated significant differences in the frequencies of several HLA alleles between patients with IgAN and the control group. This is also the first study to investigate the link between genetic variations and pathological findings in a Taiwanese cohort. However, we did not find a robust association between HLA alleles and MEST-C features after rigorous multivariable adjustment. However, no HLA AFs were associated with progression to ESKD or early CKD. Continued genetic research is essential to elucidate the role of HLA in the pathogenesis of IgAN and to explore the potential of genetic studies in improving diagnosis and prognosis prediction in patients with IgAN.

## Figures and Tables

**Table 1:** Characteristics of the Study Participants.

Variable ^a^	With IgA Nephropathy (*n* = 157)	Without IgA Nephropathy (*n* = 1570)	*p*-Value ^b^
Demographics			
Age (years), mean ± SD	47.0 ± 14.0	47.0 ± 14.0	1.000
Follow-up time (years), mean ± SD	7.67 ± 2.92	–	–
Female, *n* (%)	77 (49.0)	770 (49.0)	1.000
Comorbidities, *n* (%)			
Hyperlipidemia	72 (45.9)	464 (29.6)	<0.001
Hypertension	59 (37.6)	403 (25.7)	0.002
Diabetes mellitus	25 (15.9)	386 (24.6)	0.020
Chronic obstruction pulmonary disease	2 (1.3)	44 (2.8)	0.382
Rheumatoid arthritis	0 (0.0)	16 (1.0)	0.404
Ankylosing spondylitis	1 (0.6)	31 (2.0)	0.382
Laboratory data (serum), mean ± SD			
BUN (mg/dL)	30.4 ± 28.3	27.5 ± 26.0	0.196
Creatinine (mg/dL)	2.7 ± 3.6	2.2 ± 3.4	0.063
eGFR (mL/min/1.73 m^2^)	56.2 ± 33.8	83.5 ± 36.6	<0.001
Albumin (g/dL)	4.0 ± 0.4	4.2 ± 0.5	<0.001
Uric acid (mg/dL)	6.8 ± 2.1	5.9 ± 1.8	<0.001
Hemoglobin (g/dL)	12.5 ± 2.3	13.0 ± 2.3	0.005
IgA (mg/dL)	344.3 ± 162.7	261.1 ± 269.4	<0.001
Laboratory data (urine)			
Protein/creatinine ratio (mg/g)	1506.9 ± 2302.8	–	–
Medication use, *n* (%)			
ACE inhibitor	10 (6.4)	46 (2.9)	0.037
ARB	136 (86.6)	448 (28.5)	<0.001
Systemic corticosteroid	96 (61.2)	378 (24.1)	<0.001
Calcineurin inhibitor	43 (27.4)	208 (13.3)	<0.001
Mycophenolate mofetil	23 (14.7)	179 (11.4)	0.281
SGLT2 inhibitor	6 (3.8)	69 (4.4)	0.896

^a^ Values are expressed as mean ± standard deviation (SD) for continuous variables and as number (percentage) for categorical variables. ^b^ *p*-values were calculated using Student’s *t*-test for continuous variables and chi-square test for categorical variables. Abbreviations: eGFR: estimated glomerular filtration rate; ACE: angiotensin-converting enzyme; ARB: angiotensin II receptor blockers; SGLT2: sodium-glucose cotransporter-2.

**Table 2:** Clinical and Pathological Characteristics of Patients with IgAN.

Pathology (Oxford-MESTC Score) (*n* = 101)	*n*	%
M0	40	39.6
M1	61	60.4
E0	72	71.3
E1	28	27.7
S0	27	26.7
S1	73	72.3
T0	72	71.3
T1	18	17.8
T2	11	10.9
C0	91	90.1
C1	7	6.9
C2	2	2.0
Immunofluorescence (*n* = 117)	*n*	%
IgA deposit > 3+	62	53.0
IgG deposit positive	11	9.4
IgM deposit positive	25	21.4
C1q deposit positive	2	1.7
C3 deposit positive	89	75.4
Kappa deposit positive	86	74.1
Lambda deposit positive	103	87.3

Pathological findings according to the Oxford-MEST-C classification system (*n* = 100) and immunofluorescence patterns (*n* = 117) in patients with biopsy-proven IgA nephropathy. M, mesangial hypercellularity; E, endocapillary hypercellularity; S, segmental glomerulosclerosis; T, tubular atrophy/interstitial fibrosis; C, crescents. Data are presented as numbers (percentages).

**Table 3:** Genotype Frequencies of HLA Alleles.

Variables		Total		With IgA Nephropathy		Without IgA Nephropathy		*p*-Value ^a^	Risk of IgA Nephropathy		
		*n* = 3454 (Alleles)		*n* = 314 (Alleles)		*n* = 3140 (Alleles)					
		*n*	(%)	*n*	(%)	*n*	(%)		OR	95%CI	*p*-Value ^b^
B*58:01	no	3092	89.8	294	94.8	2798	89.3	0.003	0.46	0.27–0.76	0.036
	yes	350	10.2	16	5.2	334	10.7				
C*08:01	no	3145	91.4	267	86.1	2878	91.9	0.0008	1.83	1.29–2.58	0.001
	yes	297	8.6	43	13.9	254	8.1				
DQA1*01:05	no	3415	99.2	303	97.7	3112	99.4	0.006	3.60	1.51–8.58	0.004
	yes	27	0.8	7	2.3	20	0.6				
DQA1*03:01	no	3152	91.6	266	85.8	2886	92.2	0.0002	1.94	1.38–2.74	<0.001
	yes	290	8.4	44	14.2	246	7.9				
DQA1*03:03	no	3125	90.8	263	84.8	2862	91.4	0.0002	1.89	1.36–2.65	<0.001
	yes	317	9.2	47	15.2	270	8.6				
DQA1*05:01	no	3165	92	303	97.7	2862	91.4	0.0001	0.24	0.11–0.52	<0.001
	yes	277	8.1	7	2.3	270	8.6				
DQB1*02:01	no	3165	92	303	97.7	2862	91.4	0.00013	0.24	0.11–0.52	<0.001
	yes	277	8.1	7	2.3	270	8.6				
DRB1*03:01	no	3164	91.9	303	97.7	2861	91.4	0.0001	0.24	0.11–0.52	<0.001
	yes	278	8.1	7	2.3	271	8.7				
DQB1*03:02	no	3121	90.7	265	85.5	2856	91.2	0.00141	1.76	1.25–2.47	0.0043
	yes	321	9.3	45	14.5	276	8.8				
DQB1*04:01	no	3170	92.1	265	85.5	2905	92.8	0.00001	2.17	1.54–3.07	<0.001
	yes	272	7.9	45	14.5	227	7.3				
DRB1*04:03	no	3267	94.9	282	91	2985	95.3	0.0015	2.02	1.32–3.08	0.003
	yes	175	5.1	28	9	147	4.7				
DRB1*04:05	no	3137	91.1	263	84.8	2874	91.8	0.0001	1.99	1.42–2.79	<0.001
	yes	305	8.9	47	15.2	258	8.2				
DRB1*10:01	no	3415	99.2	303	97.7	3112	99.4	0.006	3.61	1.51–8.58	0.005
	yes	27	0.8	7	2.3	20	0.6				

^a^ Categorical variables are presented as numbers (percentages) and were analyzed using the chi-square test. ^b^ *p*-values were derived from multivariable logistic regression analysis adjusted for age, sex, hypertension, diabetes, and hyperlipidemia, and were corrected for multiple testing using the false discovery rate (FDR) method.

**Table 4:** Genotype frequencies of HLA alleles according to pathological features in patients with IgAN.

Variables			M0		M1		*p* Value ^a^	E0		E1		*p* Value ^a^	S0			S1		*p* Value ^a^
			*n* = 80 (Alleles)		*n* = 120 (Alleles)			*n* = 144 (Alleles)		*n* = 56 (Alleles)			*n* = 54 (Alleles)			*n* = 146 (Alleles)		
			*n*	(%)	*n*	(%)		*n*	(%)	*n*	(%)		*n*	(%)		*n*	(%)	
C*08:01	no		69	86.25	104	86.67	1.0000	125	86.81	48	85.71	1.0000	47	87.04		126	86.30	1.0000
	yes		11	13.75	16	13.33		19	13.19	8	14.29		7	12.96		20	13.70	
DQA1*01:05	no		78	97.50	116	96.67	1.0000	139	96.53	55	98.21	0.8680	54	100.00		140	95.89	0.2957
	yes		2	2.50	4	3.33		5	3.47	1	1.79		0	0.00		6	4.11	
DQA1*03:01	no		71	88.75	101	84.17	0.4795	123	85.42	49	87.50	0.8774	52	96.30		120	82.19	0.0202
	yes		9	11.25	19	15.83		21	14.58	7	12.50		2	3.70		26	17.81	
DQA1*03:03	no		68	85.00	102	85.00	1.0000	121	84.03	49	87.50	0.6914	46	85.19		124	84.93	1.0000
	yes		12	15.00	18	15.00		23	15.97	7	12.50		8	14.81		22	15.07	
DQB1*03:02	no		70	87.50	101	84.17	0.6520	122	84.72	49	87.50	0.7815	51	94.44		120	82.19	0.0502
	yes		10	12.50	19	15.83		22	15.28	7	12.50		3	5.56		26	17.81	
DQB1*04:01	no		69	86.25	102	85.00	0.9673	122	84.72	49	87.50	0.7815	47	87.04		124	84.93	0.8813
	yes		11	13.75	18	15.00		22	15.28	7	12.50		7	12.96		22	15.07	
DRB1*04:03	no		74	92.50	107	89.17	0.5882	128	88.89	53	94.64	0.3283	52	96.30		129	88.36	0.1531
	yes		6	7.50	13	10.83		16	11.11	3	5.36		2	3.70		17	11.64	
DRB1*04:05	no		68	85.00	101	84.17	1.0000	121	84.03	48	85.71	0.9376	46	85.19		123	84.25	1.0000
	yes		12	15.00	19	15.83		23	15.97	8	14.29		8	14.81		23	15.75	
DRB1*10:01	no		78	97.50	116	96.67	1.0000	139	96.53	55	98.21	0.8680	54	100.00		140	95.89	0.2957
	yes		2	2.50	4	3.33		5	3.47	1	1.79		0	0.00		6	4.11	
**Variables**			**T0**			**T1 or T2**			***p* Value ^a^**		**C0**			**C1 or C2**				***p* Value ^a^**
			***n* = 144 (Alleles)**			***n* = 56 (Alleles)**					***n* = 182 (Alleles)**			***n* = 18 (Alleles)**				
			***n***		**(%)**	***n***		**(%)**			***n***	**(%)**		***n***	**(%)**			
C*08:01		no	122		84.72	51		91.07	0.3424		158	86.81		15	83.33			0.9596
		yes	22		15.28	5		8.93			24	13.19		3	16.67			
DQA1*01:05		no	141		97.92	53		94.64	0.4490		177	97.25		17	94.44			1.0000
		yes	3		2.08	3		5.36			5	2.75		1	5.56			
DQA1*03:01		no	122		84.72	50		89.29	0.5431		157	86.26		15	83.33			1.0000
		yes	22		15.28	6		10.71			25	13.74		3	16.67			
DQA1*03:03		no	121		84.03	49		87.50	0.6914		152	83.52		18	100.00			0.1279
		yes	23		15.97	7		12.50			30	16.48		0	0.00			
DQB1*03:02		no	121		84.03	50		89.29	0.4687		156	85.71		15	83.33			1.0000
		yes	23		15.97	6		10.71			26	14.29		3	16.67			
DQB1*04:01		no	122		84.72	49		87.50	0.7815		153	84.07		18	100.00			0.1387
		yes	22		15.28	7		12.50			29	15.93		0	0.00			
DRB1*04:03		no	131		90.97	50		89.29	0.9230		165	90.66		16	88.89			1.0000
		yes	13		9.03	6		10.71			17	9.34		2	11.11			
DRB1*04:05		no	120		83.33	49		87.50	0.6076		151	82.97		18	100.00			0.1179
		yes	24		16.67	7		12.50			31	17.03		0	0.00			
DRB1*10:01		no	141		97.92	53		94.64	0.4490		177	97.25		17	94.44			1.0000
		yes	3		2.08	3		5.36			5	2.75		1	5.56			

^a^ Categorical variables are presented as numbers (percentages) and were analyzed using the chi-square test.

**Table 5:** Summary of the comparison between HLA alleles showing significant associations in this and the other studies.

Report	Alleles with Increased IgAN Risk	Alleles with Decreased IgAN Risk	Alleles with Risk of ESKD in IgAN	Reference
This study	C*08:01, DQA1*01:05, DQA1*03:01, DQA1*03:03, DQB1*03:02, DQB1*04:01, DRB1*04:03, DRB1*04:05, DRB1*10:01	B*58:01, DQA1*05:01, DQB1*02:01, DRB1*03:01	None	
Raguénès, 1995	B1*04, DQB1*03:01		DQB1*03:01	[42]
Fennessy, 1996		DQB1*02:01, DQB1*06:02		[35]
Cao, 2008	DRB1*14:05:01	DRB1*07:01:01	DRB1*03:01:01 (lower risk)	[37]
Yu, 2011	A*11:01, B*40:01, DQB*03:02			[32]
Jiyun, 2012	DRB1*04:03, DRB1*04:05			[43]
In, 2022	DRB1*04:05, DQB1 *04:01, DQB1*03:02	DRB1*07:01, DRB1*15:01, DQB1*02:02, DQB1*06:02	DQB1*05:03 (lower risk)	[34]

## Data Availability

The datasets generated and analyzed during the current study are not publicly available due to the policies of TPMI project but are available from the corresponding author on reasonable request.

## Supplementary Materials

The following supporting information can be downloaded at: https://www.mdpi.com/article/10.3390/ijms27020790/s1.
