# Supplementary material for: Association Between HLA Alleles and IgA Nephropathy in a Taiwanese Population
Source: Int J Mol Sci. 2026 Jan 13;27(2):790. doi: 10.3390/ijms27020790 (PMC12841225; doi:10.3390/ijms27020790)

Supplementary Materials

Supplementary Table S1. Summary of HLA imputation quality control metrics.

HLA locus	Mean call rate (%)	Concordance with reference typing (%)
**HLA**	98.7	97.8
**HLB**	98.2	98.1
**HLC**	97.0	97.5
**HLA-DPA1**	98.9	98.4
**HLA-DPB1**	98.5	97.9
**HLA-DQA1**	99.2	98.3
**HLA-DQB1**	99.1	97.9
**HLA-DRB1**	95.4	91.2

Supplementary Table S2. Association between HLA alleles and the presence of segmental glomerulosclerosis in patients with IgA nephropathy.

HLA alleles	Risk of segmental glomerulosclerosis		
	OR	95%CI	*P*-value[a]
**C*08:01**	1.16	0.46-2.96	0.94
**DQA1*01:05**	NA	-	-
**DQA1*03:01**	5.56	1.26-24.48	0.16
**DQA1*03:03**	1.07	0.44-2.60	0.93
**DQB1*03:02**	3.58	1.03-12.45	0.24
**DQB1*04:01**	1.27	0.50-3.22	0.92
**DRB1*04:03**	3.30	0.73-14.98	0.28
**DRB1*04:05**	1.11	0.46-2.69	0.93
**DRB1*10:01**	NA	-	-

[a] P-values were derived from multivariable logistic regression analysis adjusted for age, sex, hypertension, diabetes, and hyperlipidemia, and were corrected for multiple testing using the BH-FDR method.For alleles DQA1*01:05 and DRB1*10:01, no carriers were present in the S0 group; therefore, odds ratios (ORs) and confidence intervals (CIs) could not be estimated (NA).

Supplementary Table S3. Distribution of HLA Allele Genotypes by ESKD and Early CKD Status

Variables		Non-ESKD n=160		ESKD n=124		*p* value[a]	Non-ESKD n=160		ESKD and early CKD n=148		*p* value[a]
		n	(%)	n	(%)		n	(%)	n	(%)	
**C*08:01**	**no**	140	87.50	104	83.87	0.4839	140	87.50	125	84.46	0.5454
	**yes**	20	12.50	20	16.13		20	12.50	23	15.54	
**DQA1*01:05**	**no**	157	98.12	122	98.39	1.0000	157	98.12	144	97.30	0.9169
	**yes**	3	1.88	2	1.61		3	1.88	4	2.70	
**DQA1*03:01**	**no**	140	87.50	105	84.68	0.6089	140	87.50	125	84.46	0.5454
	**yes**	20	12.50	19	15.32		20	12.50	23	15.54	
**DQA1*03:03**	**no**	140	87.50	103	83.06	0.3764	140	87.50	121	81.76	0.2143
	**yes**	20	12.50	21	16.94		20	12.50	27	18.24	
**DQB1*03:02**	**no**	139	86.88	105	84.68	0.7218	139	86.88	125	84.46	0.6583
	**yes**	21	13.12	19	15.32		21	13.12	23	15.54	
**DQB1*04:01**	**no**	142	88.75	103	83.06	0.2275	159	99.38	145	97.97	0.5605
	**yes**	18	11.25	21	16.94		1	0.62	3	2.03	
**DRB1*04:03**	**no**	150	93.75	109	87.90	0.1301	150	93.75	130	87.84	0.1085
	**yes**	10	6.25	15	12.10		10	6.25	18	12.16	
**DRB1*04:05**	**no**	141	88.12	102	82.26	0.2206	141	88.12	120	81.08	0.1190
	**yes**	19	11.88	22	17.74		19	11.88	28	18.92	
**DRB1*10:01**	**no**	157	98.12	122	98.39	1.0000	157	98.12	144	97.30	0.9169

**yes**	3	1.88	2	1.61	3	1.88	4	2.70

Supplementary Figure S1. Flow diagram of study participant selection.

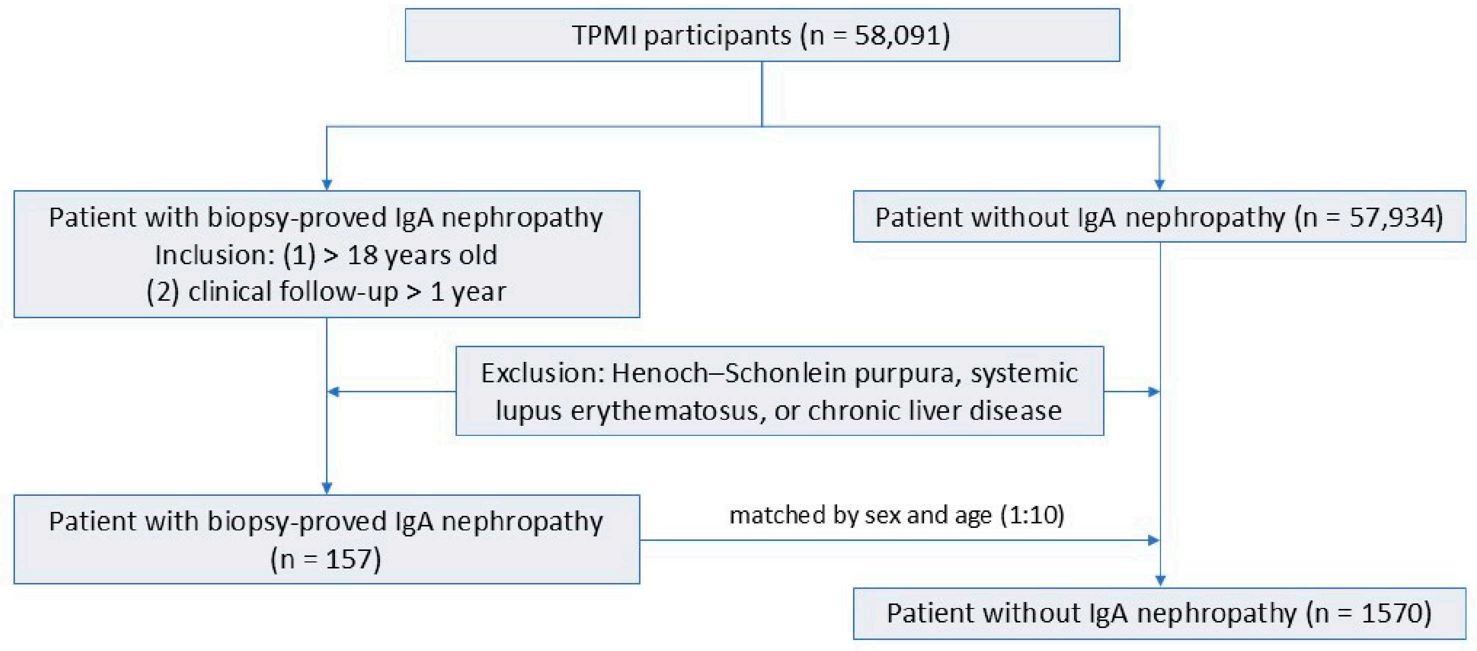

Supplement: Supplementary file 1 [file ijms-27-00790-s001.zip › ijms-3866607-supplementary.pdf]
